# Traumatic posterior shoulder dislocation with associated acromion fracture: a report of 2 cases

**DOI:** 10.1016/j.xrrt.2025.09.006

**Published:** 2025-09-18

**Authors:** Alexander J. Vervaecke, Alain Meyer, Bastian Sigrist, Christian Gerber, Jean-David Werthel

**Affiliations:** a Department of Orthopaedic Surgery, Hôpital Ambroise-Paré, Boulogne-Billancourt, France; b Monica Orthopaedic Research (MoRe) Foundation, Antwerp, Belgium; c Clinique du Sport Paris V, Paris, France; d Balgrist Campus Research and Development Center, Zürich, Switzerland

**Keywords:** Acromion fracture, Posterior shoulder instability, Shoulder dislocation, Static posterior subluxation, Shoulder instability, Acromial morphology

Traumatic posterior shoulder dislocations are rare, comprising 2% to 5% of all shoulder dislocations. They typically result from high-energy trauma, such as sports injuries, motor vehicle accidents, or seizures.^13^ Associated injuries, including glenoid and/or humeral fractures or rotator cuff tears, occur in up to 65% of cases.^9^^,^^12^

Certain anatomical features may predispose shoulders to traumatic posterior shoulder dislocations. A matched-control study identified significant associations between posterior shoulder instability and increased glenoid retroversion or dysplasia.^4^ However, the relationship between scapular anatomy and posterior glenohumeral stability extends beyond the glenoid. In 2019, Meyer et al highlighted the role of acromial morphology in posterior instability.^10^ Specifically, a posterior acromion that is higher and more flat offers less posterior osseous restraint, predisposing to posterior instability. Conversely, a lower, more oblique acromion, typical in healthy shoulders and in cases of anterior shoulder instability, acts as a protective buttress against posterior displacement.^7^^,^^8^

We present 2 cases of traumatic posterior shoulder dislocations associated with fractures of the acromion and the posterior glenoid rim. While posterior shoulder dislocation most often occurs without acromial injury, we hypothesize that in certain shoulders, a fracture of the posterior acromion appears necessary for a complete posterior glenohumeral dislocation to occur.

## Case report

### Case 1

**Figure 1:**
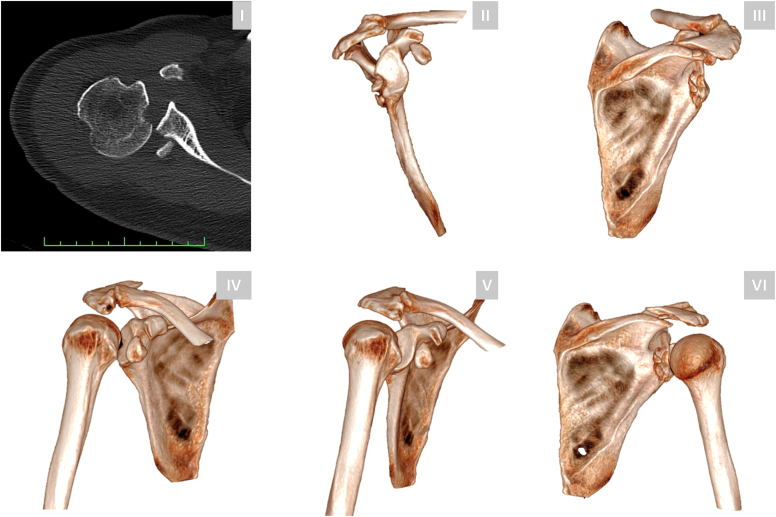
Preoperative CT imaging of a 61-year-old male following a high-velocity motor bicycle accident. Axial CT image (**I**) and preoperative 3D reconstructions (**II-VI**) provide a detailed visualization of the complex injury pattern, including an acromion fracture, posterior shoulder dislocation, posterior glenoid rim fracture, and coracoid fracture. *CT*, computed tomography; *3D*, three-dimensional.

A 61-year-old right-hand-dominant male sustained a right shoulder injury following a high-velocity motor bicycle accident. Neurological and vascular examinations were intact. Computed tomography (CT) imaging displayed (1) a posterior glenohumeral dislocation, (2) an acromion fracture, (3) a coracoid fracture (Eyres type II), (4) a posterior Bankart fracture, and (5) a reverse Hill-Sachs lesion (Fig. 1). Closed reduction under fluoroscopy was attempted; however; it failed due to persistent instability and recurrent dislocation. The patient was subsequently referred to our institution for surgical management.

The patient was taken to the operating room, positioned in a lateral decubitus position, and an L-shaped incision was made along the scapular spine and posterior deltoid border. A Brodsky deltoid-sparing approach exposed the interval between the infraspinatus and teres minor.^3^ The posterior glenohumeral capsule was incised vertically to access the posterior bony Bankart fracture. The fragment was severely comminuted and therefore not amenable to stable fixation, so it was excised. The glenoid neck was prepared, and a 26 mm × 10 mm tricortical iliac crest autograft was shaped as a posterior bone block, positioned flush with the articular glenoid cartilage, and fixed with 2 4.5 mm cannulated self-tapping titanium screws. Subsequently, the posterior labrum was repaired using 3.0 polydioxanone sutures.

To address the scapular spine fracture, the posterior deltotrapezoid fascia was incised, and the trapezius insertion was subperiosteally elevated along the fracture line. The fracture was débrided and satisfactorily reduced under visual control. An olecranon 3.5 mm locking plate was contoured to hook the acromion as described by Ting et al and placed dorsally along the scapular spine.^15^ The plate was secured with bicortical screws, and the posterior deltotrapezoid fascia was closed. The coracoid process fracture was managed conservatively.

**Figure 2:**
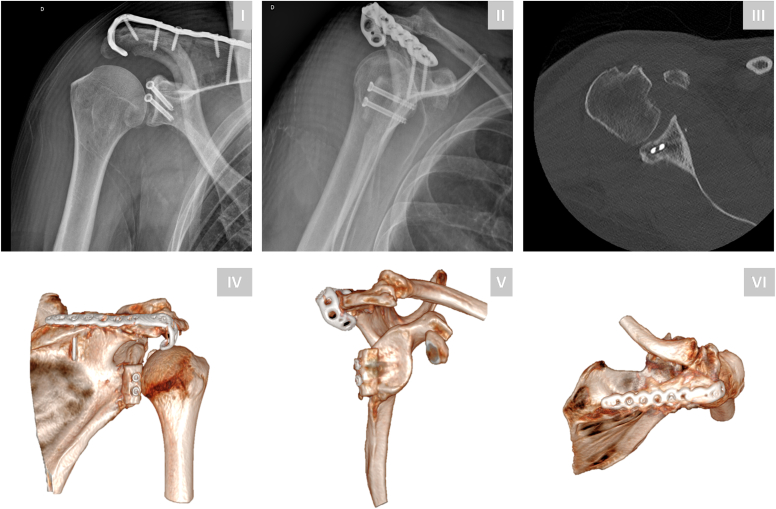
Postoperative imaging following open reduction, posterior glenoid bone block placement, and plate fixation of the acromion fracture. Standard anteroposterior and lateral radiographs (**I-II**) demonstrate appropriate reduction and implant placement. Axial CT imaging (**III**) and 3D reconstructions (**IV-VI**) confirm reduction and centering of the humeral head. *CT*, computed tomography; *3D*, three-dimensional.

Immediate postoperative radiographs and CT scans confirmed recentering of the humeral head and appropriate implant positioning (Fig. 2). The patient was immobilized in an abduction brace for 4 weeks, followed by a structured rehabilitation program consisting of progressive passive and active range of motion (ROM) exercises. Strengthening was allowed at 3 months postoperatively.

**Figure 3:**
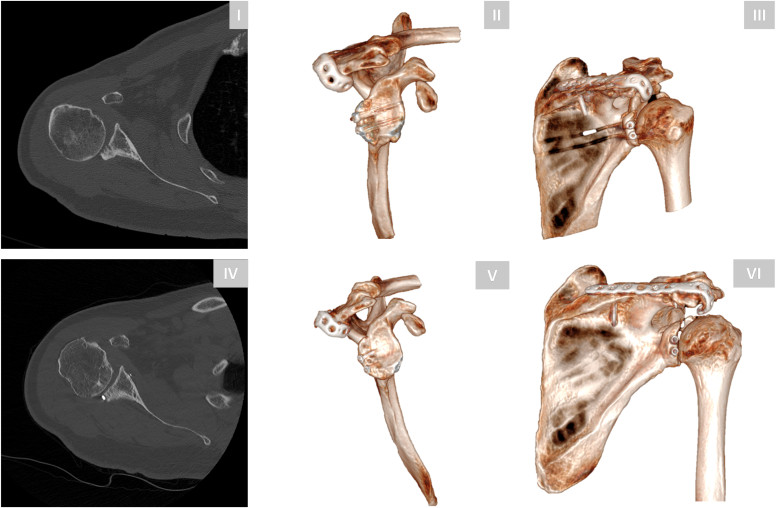
Postoperative CT imaging with 3D reconstructions at 1-year (**I-III**) and 3-year (**IV-VI**) follow-up. Imaging confirms complete consolidation of the acromion fracture and posterior bone block. The coracoid process fracture remained ununited and progressed into an asymptomatic nonunion. Progressive static posterior subluxation was observed at 1-year follow-up and persisted at final follow-up, with mild progression of glenohumeral osteoarthritis. *CT*, computed tomography; *3D*, three-dimensional.

**Figure 4:**
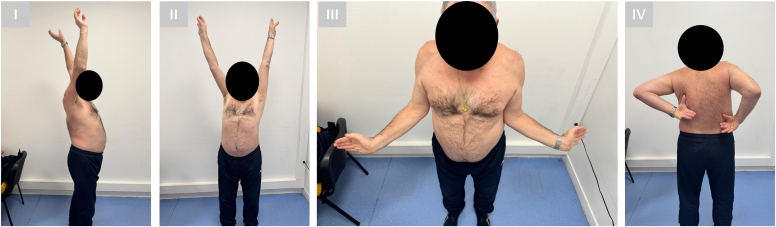
Clinical evaluation of active range of motion at final follow-up, 3 years postoperatively. (**I**) Anterior elevation of 160°, (**II**) abduction to 150°, (**III**) external rotation in adducted position of 60°, and (**IV**) functional internal rotation reaching the L3-L1 level.

At 1-year follow-up, CT imaging demonstrated healing of the acromion fracture and bone block. However, progressive static posterior subluxation of the humeral head was observed (Fig. 3). The coracoid fracture progressed to asymptomatic nonunion. The patient exhibited no signs of posterior apprehension clinically. At final follow-up, 3 years postoperatively, imaging revealed persistent static posterior subluxation with mild progression of glenohumeral osteoarthritis (Fig. 3). Clinically, a minor loss of active end-ROM was observed, particularly in internal rotation (Fig. 4). Despite these limitations, the patient reported satisfactory function with a subjective shoulder value of 80%, Constant score of 78, and an American Shoulder and Elbow Surgeons score of 70.

### Case 2

**Figure 5:**
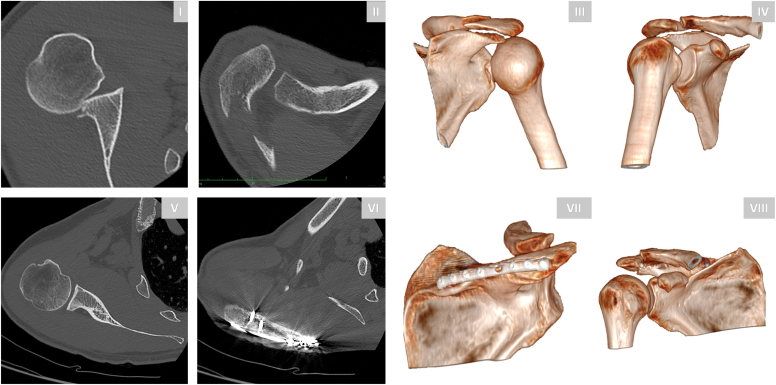
Preoperative and postoperative CT imaging and 3D reconstructions of a 36-year-old male with a posterior shoulder dislocation and acromion fracture. Preoperative images (**I-IV**) illustrate the combined dislocation and fracture pattern and associated reverse Hill-Sachs impaction fracture. Postoperative images (**V-VIII**) confirm bony consolidation of the acromion fracture and correct centering of the humeral head relative to the scapular axis. *CT*, computed tomography; *3D*, three-dimensional.

A 36-year-old right-hand-dominant male sustained a right shoulder injury with a fracture pattern comparable to the previous case following a motor vehicle accident. Neurological and vascular functions were intact. CT imaging revealed a posterior shoulder dislocation with a reverse Hill-Sachs lesion and acromion fracture (Fig. 5).

Under general anesthesia, closed reduction was performed, and the patient was positioned in a beach chair position. A horizontal incision overlying the scapular spine was made, and the fracture site was exposed. The fracture was reduced and stabilized using a dorsal 3.5 mm locking plate with bicortical screws. Intraoperative dynamic testing of the glenohumeral joint under fluoroscopy demonstrated a stable joint without engagement of the reverse Hill-Sachs lesions, which was therefore managed nonsurgically.

Postoperatively, the patient was immobilized in an abduction brace in neutral rotation for 4 weeks, followed by progressive motion with physiotherapy. By four months postoperatively, the patient had returned to work as a delivery truck driver with near-symmetrical ROM. CT imaging at 1-year follow-up confirmed uneventful fracture healing with no residual posterior subluxation (Fig. 5). Clinical testing demonstrated a subjective shoulder value of 75%, American Shoulder and Elbow Surgeons score of 83, and Constant score of 67%.

## Discussion

Traumatic posterior shoulder dislocations are uncommon but frequently present with associated fractures or rotator cuff tears.^12^ The fractures commonly involve the proximal humerus (neck, tuberosities, or impaction fractures), and less frequently the posterior glenoid rim.^12^ We present 2 cases of traumatic posterior shoulder dislocation associated with acromion fractures. This combined injury pattern was first described in one case in 2003, and to the best of our knowledge, no subsequent mention of this lesion was reported.^6^ While posterior shoulder dislocations commonly occur in the absence of acromial injury, these cases raise the question of the potential importance of acromial morphology in the pathomechanics of posterior dislocation.

Previous studies have suggested that specific scapular anatomy predisposes patients to static and recurrent dynamic posterior instability. Glenoid retroversion or dysplasia, as well as a high and flat posterior acromion, can increase the risk of posterior dislocations.^10^ This occurs because a high, flat acromion lacks the posterior osseous buttress typically provided by a lower, more oblique acromion. In the cases presented, we can only explain the acromion fractures resulting from the significant posterior force exerted by the humeral head during displacement. Although preinjury scapulometric data of these shoulders were unavailable, we postulated that the applied force likely exceeded the stability of the posterior acromion, compromising its restraining function and leading to a fracture with subsequent posterior dislocation.

In the first case, surgical intervention was chosen to address both the posterior glenoid bone loss and the acromion fracture, as the patient demonstrated recurrent instability following closed reduction. Posterior bone block procedures are typically used in cases of structural instability where posterior glenoid bone loss exceeds 10%, as they serve to enlarge the posterior glenoid track.^1^^,^^14^ To restore the posterior buttress, surgical fixation of the acromion was performed. The plate was positioned dorsally along the subcutaneous border of the scapular spine, complemented by a hook construct under the lateral acromion. This approach was selected based on biomechanical analyses suggesting superior stability with this configuration.^15^ The chosen technique achieved bony consolidation without recurrence of dynamic dislocation or apprehension, resulting in a satisfactory functional outcome for the patient.

**Figure 6:**
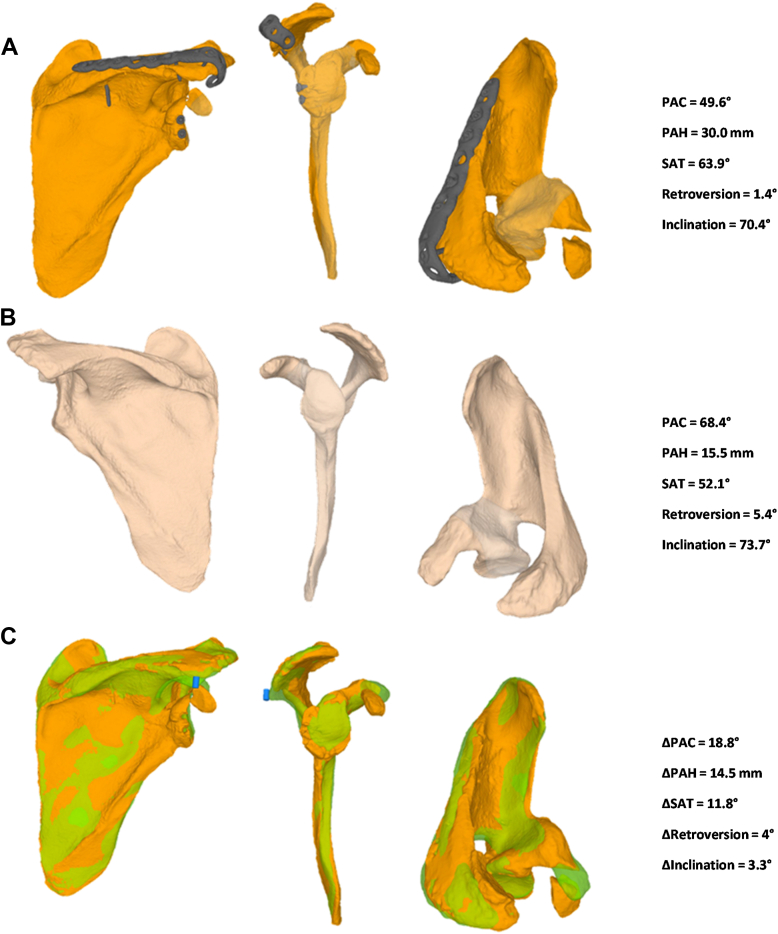
Morphometric 3D comparison of the injured scapula of case 1 with the contralateral healthy scapula based on CT imaging at 3-year follow-up. The analysis demonstrated that the acromion healed in a malreduced, higher and more horizontally orientated position (**A**), providing insufficient posterior coverage of the humeral head when compared to the uninjured side (**B**). Quantitative assessment revealed a notable reduction in posterior acromial coverage (PAC), increase in posterior acromial height (PAH) and increase in sagittal acromial tilt (SAT) relative to the contralateral scapula. Overlay images (**C**) of the injured scapula (*orange*) and mirrored healthy side (*green*) provide a direct visualization of the positional differences. *CT*, computed tomography; *3D*, three-dimensional.

However, the patient progressed toward static posterior subluxation of the humeral head. This could potentially be attributed to inadequate reduction of the acromion, which may have failed to sufficiently restore the posterior coverage of the humeral head.^2^ To investigate this hypothesis, a morphometric three-dimensional (3D) comparison of the injured shoulder with the contralateral healthy scapula was conducted as previously described (Fig. 6).^5^^,^^10^ The analysis revealed a 31.9% reduction in posterior acromial coverage, measuring 49.6° in the injured scapula compared to 68.4° in the uninjured side (Δ18.8°). The posterior acromial height increased by 63.7%, measuring 30.0 mm in the injured scapula versus 15.5 mm in the contralateral side (Δ14.5 mm). Additionally, the sagittal acromial tilt increased by 20.3%, measuring 63.9° compared to 52.1° on the uninjured scapula (Δ11.8°).

These findings suggest that the acromion healed in a malreduced, higher, and more horizontally orientated position, providing insufficient posterior coverage of the humeral head when compared to the uninjured side. Initial imaging confirmed a centered humeral head, suggesting that passive alignment was restored; however, this does not exclude the possibility of biomechanical imbalance. It may reflect a temporary equilibrium that was later lost. In this case, we postulated that the posterior bone block initially acted as a stabilizing element, compensating for the acromial morphology. Its progressive remodeling may have unmasked the biomechanical insufficiency of the malreduced acromion, ultimately contributing to the observed static subluxation. Furthermore, the divergent values observed in this case were more pronounced than the mean scapular morphometric values reported in a cohort of 41 shoulders with static posterior dislocations (Table I).^10^ The severe acromial malalignment could therefore explain the development of posterior static subluxation despite the successful restoration of the glenoid retroversion through the bone block procedure.

**Table I:** Comparison of scapular morphometric data from the 2 presented cases with mean values and standard deviations reported in the cohort study performed by Meyer et al.

Scapula measurements	Case 1 (injured shoulder)	Case 2 (injured shoulder)	Posterior static Subluxation group (Meyer et al)^5^	Healthy shoulder Control group (Meyer et al)^5^
Posterior acromial coverage (PAC)	49.6°	66.5°	54.6° ± 6.7°	62.9° ± 7.5°
Posterior acromial height (PAH)	30.0 mm	18.4 mm	21.3 mm ± 4.2 mm	15.5 mm ± 4.9 mm
Sagittal acromial tilt (SAT)	63.9°	62.3°	63.0 ± 8.5°	55.7 ± 7.6

This study included patients with posterior static subluxation (n = 41 shoulders) and a healthy control group without instability or degenerative glenohumeral changes (n = 53 shoulders).

**Figure 7:**
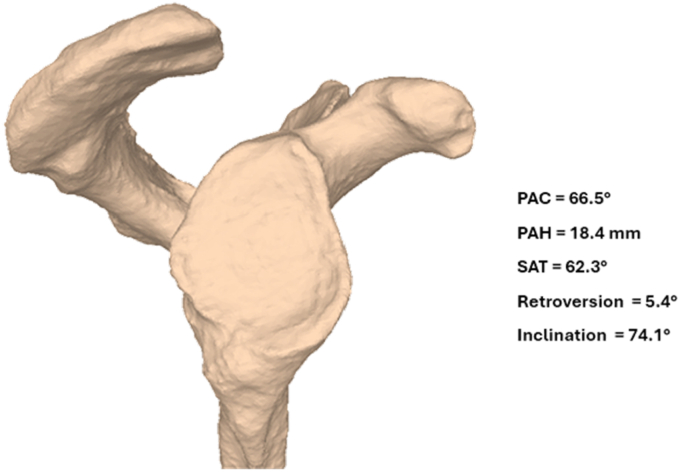
Morphometric 3D analysis of the injured scapula in case 2 at 1-year follow-up. The assessment demonstrates adequate reduction of the acromial position with successful restoration of posterior acromial coverage (PAC) and posterior acromial height (PAH). *SAT*, sagittal acromial tilt. *3D*, three-dimensional.

In the second case, glenoid bone loss was less pronounced, measuring 11.2% compared to the 31.8% in the first case when quantified using the best-fit circle method on 3D reconstructed en-face views. The dislocation was managed with closed reduction, and following fixation of the acromion, the joint remained stable throughout the free ROM, obviating the need for additional surgical intervention.^11^ Postoperative 3D morphometric analysis confirmed proper acromial reduction and restoration of posterior acromial coverage and posterior acromial height (Fig. 7) corresponding to the mean values of healthy control shoulders as reported by Meyer et al (Table I).^5^ No static subluxation of the glenohumeral joint occurred throughout the observation period.

## Conclusion

Two cases document an unusual injury pattern in which a posterior glenohumeral dislocation occurred in association with a (posterior) acromion fracture. In select shoulders with a normally positioned and oriented acromion, for a posterior dislocation to occur, high-energy trauma may be able to exert sufficient force to fracture the acromion. In such cases, anatomical reduction and retention of the acromion fracture may be needed to prevent recurrent posterior instability.

## Disclaimers:

Funding: No funding was disclosed by the authors.

Conflicts of interest: Jean-David Werthel serves as a consultant and receives royalties for shoulder arthroplasty implants from Stryker. The other authors, their immediate families, and any research foundations with which they are affiliated have not received any financial payments or other benefits from any commercial entity related to the subject of this article.

Patient consent: The authors confirm that both patients were informed and agreed for their anonymized data to be submitted for publication.
